# Placental transcriptome analysis of hypertensive pregnancies identifies distinct gene expression profiles of preeclampsia superimposed on chronic hypertension

**DOI:** 10.1186/s12920-023-01522-x

**Published:** 2023-05-02

**Authors:** Ashley M. Hesson, Elizabeth S. Langen, Olesya Plazyo, Johann E. Gudjonsson, Santhi K. Ganesh

**Affiliations:** 1 Division of Maternal Fetal Medicine, Department of Obstetrics and Gynecology, University of Michigan, 1500 East Medical Center Dr., Ann Arbor, MI 48109 USA; 2 Departments of Dermatology and Immunology, University of Michigan, Ann Arbor, MI 48109 USA; 3 Division of Cardiovascular Medicine, Department of Internal Medicine, Department of Human Genetics, University of Michigan, MSRB III / Room 7220A, 1150 West Medical Center Dr., Ann Arbor, MI 48109 USA

**Keywords:** Biomarkers, Inhibin, Preeclampsia, RNASeq, Hypertension

## Abstract

**Background:**

The pathogenesis of preeclampsia superimposed on chronic hypertension (SI) is poorly understood relative to preeclampsia (PreE) occurring in pregnant people without chronic hypertension. Placental transcriptomes in pregnancies complicated by PreE and SI have not been previously compared.

**Methods:**

We identified pregnant people in the University of Michigan Biorepository for Understanding Maternal and Pediatric Health with hypertensive disorders affecting singleton, euploid gestations (N = 36) along with non-hypertensive control subjects (N = 12). Subjects were grouped as: (1) normotensive (N = 12), (2) chronic hypertensive (N = 13), (3) preterm PreE with severe features (N = 5), (4) term PreE with severe features (N = 11), (5) preterm SI (N = 3), or (6) term SI (N = 4). Bulk RNA sequencing of paraffin-embedded placental tissue was performed. The primary analysis assessed differential gene expression relative to normotensive and chronic hypertensive placentas, where Wald adjusted *P* values < 0.05 were considered significant. Unsupervised clustering analyses and correlation analyses were performed between conditions of interest, and a gene ontology was constructed.

**Results:**

Comparing samples from pregnant people with hypertensive diseases to non-hypertensive controls, there were 2290 differentially expressed genes. The log2-fold changes in genes differentially expressed in chronic hypertension correlated better with term (R = 0.59) and preterm (R = 0.63) PreE with severe features than with term (R = 0.21) and preterm (R = 0.22) SI. A relatively poor correlation was observed between preterm SI and preterm PreE with severe features (0.20) as well as term SI and term PreE with severe features (0.31). The majority of significant genes were downregulated in term and preterm SI versus normotensive controls (92.1%, N = 128). Conversely, most term and preterm PreE with severe features genes were upregulated compared to the normotensive group (91.8%, N = 97). Many of the upregulated genes in PreE with the lowest adjusted *P* values are known markers of abnormal placentation (e.g., PAAPA, KISS1, CLIC3), while the downregulated genes with the greatest adjusted *P* values in SI have fewer known pregnancy-specific functions.

**Conclusions:**

We identified unique placental transcriptional profiles in clinically relevant subgroups of individuals with hypertension in pregnancy. Preeclampsia superimposed on chronic hypertension was molecularly distinct from preeclampsia in individuals without chronic hypertension, and chronic hypertension without preeclampsia, suggesting that preeclampsia superimposed on hypertension may represent a distinct entity.

**Supplementary Information:**

The online version contains supplementary material available at 10.1186/s12920-023-01522-x.

## Introduction

Superimposed preeclampsia on chronic hypertension (SI) presents a substantive clinical challenge in terms of its diagnosis and management [1]. Increasing anti-hypertensive needs in pregnancy can be a benign maladaptation to physiologic changes or a harbinger of multi-end organ damage requiring expedient delivery. We currently lack objective diagnostic markers to differentiate these scenarios [2]. Current clinical definitions based upon presenting history, blood pressure, and laboratory values (“severe features” [3]) are imprecise, as pregnant people with periodically severe-range blood pressures in early gestation or preexisting end-organ damage can be easily misclassified. Moreover, clinicians are currently unable to prospectively differentiate those individuals with chronic hypertension who will and will not develop adverse obstetric outcomes based on the existing clinical taxonomy [4]. As a consequence of this ambiguity, management strategies can create undue risk for some pregnant people and unnecessary burden for others: studies have shown that while rates of fetal growth restriction, abruption, and stillbirth are similar between SI and non-superimposed preeclampsia (PreE), sequelae of intervention (e.g. cesarean birth rates, neonatal intensive care intensive care admission) are more common with SI [5].

Placental transcriptomics has recently emerged as a means by which to explore differences in biologic activity across hypertension phenotypes in pregnancy. In order to more precisely define placental characteristics of PreE and SI versus uncomplicated gestations, the current study applies RNASeq analysis to placentas affected by different clinical sub-types of hypertensive pregnancy. Given the clinical importance of the presence of severe features in PreE, our analysis focuses on the contrast between SI and PreE with severe features (SF). The goal of this study, performed on paraffin embedded placental samples, is to ascertain whether or not there are transcriptomic differences in these clinically defined disease sub-classifications worthy of further investigation via more specific forms of next generation sequencing or through prospective recruitment of maternal or fetal samples. Thus, the results of the current study may contribute to the identification of potential diagnostic and therapeutic targets for those most at risk of significant adverse perinatal outcomes.

## Materials and methods

### Study subjects

The samples for this retrospective observational study were drawn from the University of Michigan Biorepository for Understanding Maternal and Pediatric Health (BUMP). This prospective biorepository collected serum, vaginal swabs, urine, umbilical cord blood, and placental segments from pregnant volunteers. In order to enroll in BUMP, participants had to be at least eighteen years of age, English speaking, and receiving care at the University of Michigan Von Voigtlander Women’s Hospital. The recruitment methods and data/sample storage protocols for BUMP were approved by an Institutional review Board (HUM# 00118179), as were the secondary analyses reported in this study (HUM# 00170182).

**Fig. 1:**
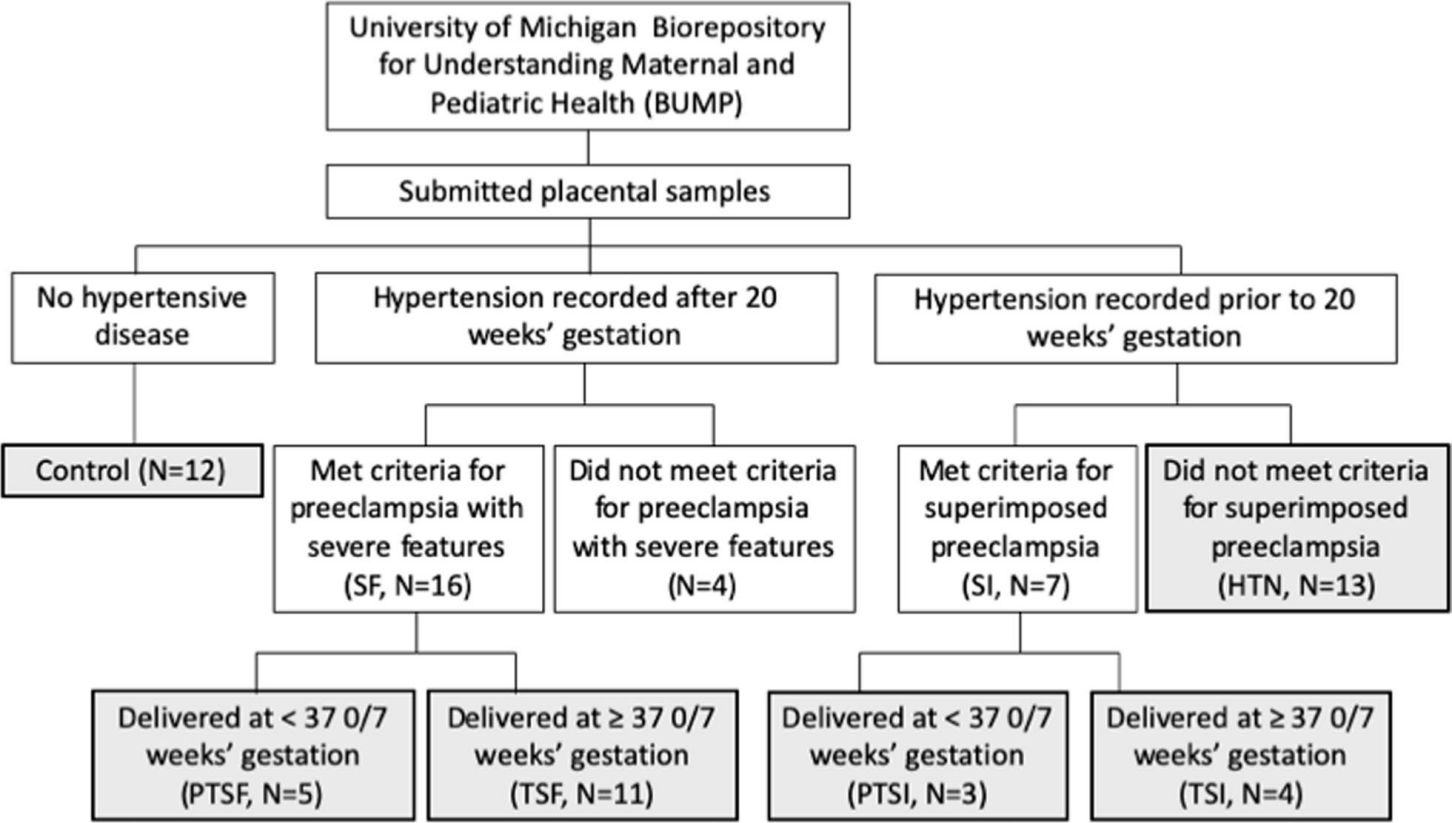
Flow diagram showing the subject-level group comparisons. Shading and bolding represent the final sub-samples included in the analysis

BUMP participants who submitted placental segments from euploid singleton gestations affected by hypertensive diseases of pregnancy and/ or chronic hypertension were sampled along with healthy controls. The BUMP samples used in this study were collected from 2017 to 2019. During this timeframe, prior to the COVID-19 pandemic, collection procedures and team members were standardized for all collections, including those for hypertensive samples and controls. Patients’ diagnoses of chronic hypertension, preeclampsia with severe features, and preeclampsia superimposed on chronic hypertension were independently verified for each case by an Obstetrician via chart review. Severe features were defined per the ACOG Task Force report on hypertension in pregnancy [3]. SI was diagnosed in the setting of new onset proteinuria or the development of severe features in a patient with documented hypertension. Figure 1 summarizes subject selection and subgroups.

### Placenta samples

Placental segments were collected from the central aspect of the disc, near the cord insertion, in full thickness cubes. Placenta samples were formalin-fixed and paraffin-embedded; five 20-micron sections of each placenta sample were subjected to RNA extraction using the RNAstorm™ kit (Cell Data Sciences, Fremont, CA, USA) as per the manufacturer’s instructions. Total RNA was processed for high-throughput sequencing using the QuantSeq 3′ mRNA-Seq Library Prep Kit FWD for Illumina (Lexogen, Greenland, NH, USA). Following mRNA generation, fragmentation, and cDNA synthesis, adaptor barcodes were ligated and final cDNA libraries were amplified by PCR and assessed for quality and concentration. Sequencing was performed using Illumina next generation platform at the University of Michigan Advanced Genomics Core.

An index of the hg38 reference genome was created and sequences were aligned to this index using the Rsubread package from the Bioconductor suite in R (Vienna, Austria). The alignment files were subsequently summarized and associated with their respective features and meta-data. Initial quality control was performed in MultiQC [6]. A 76.1% alignment was achieved for a mean of 11.0 million sequences per sample with a 36.4% GC. Phred scores for all sequenced samples are shown in Additional file 1: Item 1. Of the 82 samples submitted for sequencing, 11 were excluded from further analysis based on a priori criteria (e.g., multiple gestations, aneuploidy) and 3 were excluded for technical reasons (e.g., one was an unintended duplicate and two failed alignment).

The remaining samples’ non-normalized read counts were processed and analyzed with DESeq2 [7]. Read counts less than five were removed. Clustering analysis was performed and the results were examined to identify potential confounding variables. The following variables were considered: maternal age (less than 35 years vs greater than 35 years), maternal race (Black, White, or other), maternal autoimmune disease (presence vs absence), smoking status (smoker vs non-smoker at the time of pregnancy), BMI (< 30 vs ≥ 30), parity (nulliparous vs multiparous), estimated gestational age (term [≥ 37 completed gestational weeks] vs preterm [< 37 completed gestational weeks]), clinical chorioamnionitis (presence vs absence), and post-partum hemorrhage (estimated blood loss ≥ 1L vs estimated blood loss < 1L). Principal component analyses demonstrated well-defined clusters of individuals who smoked versus did not smoke in pregnancy, as well as those with and without auto-immune disease diagnoses (see Additional file 1: Items 2 and 3). Participants who smoked (N = 5) and had diagnosed auto-immune conditions (N = 14) were therefore excluded from further analysis to avoid confounding with our primary variable (hypertensive disease state). A final hierarchical heatmap of the 49 remaining samples identified one outlier (see Additional file 1: Item 4) that was removed due to its anomalous expression patterns relative to all other samples. This exclusion was supported by a principle component analysis that identified a dimension of variance specific to the sample in question. The final analysis included 48 samples remaining after quality control: 12 controls, 13 with chronic hypertension, 4 with term preeclampsia superimposed on chronic hypertension, 3 with preterm preeclampsia superimposed on chronic hypertension, 11 with term preeclampsia with severe features, and 5 with preterm preeclampsia with severe features (Fig. 1). There was no patterned variance in these samples based on time of collection (Additional file 1: Item 5).

The preeclamptic disease states (SI and SF) were compared to control samples and chronic hypertensive samples, where differences in gene expression were summarized in log2-fold change. Adjusted *P* values are reported by gene based on the Wald test statistic. Relationships between hypertensive disease states were assessed with clustering and correlational analyses. Functions of the top twenty most significant genes in the categories of chronic hypertension, term SF, preterm SF, term SI, and preterm SI were manually annotated using the DAVID Bioinformatics Database gene ontologies [8]. An automated gene ontology analysis was also performed using the Biological Processes ontology within ClueGO [9], an application for the visualization of functional pathways. Genes with a base-mean less than 10 and a log-fold-change less than 1 were excluded from this analysis to prevent overrepresentation of low read count and poorly differentiated genes respectively. Significance for pathway identification was set to *P* < 0.05.

## Results

### Subject characteristics

There were 48 pregnant people and associated pregnancies represented by placental samples retained for analysis (see Table 1). The mean age of participants at the time of consent was 30.9 years (SD = 5.6 years). The majority of the sample self-identified as White (72.9%, N = 35). The mean gestational age at the time of delivery was 263.4 days (37.6 weeks, SD = 19.1 days), where 81.2% (N = 39) participants delivered at term. Approximately half of the sample had a cesarean birth (52.1%, N = 25) and only 2 individuals had fetal growth restriction identified by ultrasound (4.2%).

**Table 1:** Demographic characteristics of the subjects with hypertensive pregnancies included in the RNASeq analysis

Parameter	Value	Total sample (%) (N/N_Total_)	Control (%) (N/N_Total_)	HTN (%) (N/N_Total_)	SF (%) (N/N_Total_)	SI (%) (N/N_Total_)
Parity	Primiparous	31.3 (15/48)	41.7 (5/12)	15.4 (2/13)	37.5 (6/16)	28.6 (2/7)
Age	Advanced maternal age	25.0 (12/48)	25.0 (3/12)	38.5 (5/13)	12.5 (2/16)	28.6 (2/7)
Race	Black	8.3 (4/48)	8.3 (1/12)	15.4 (2/13)	6.3 (1/16)	0. (0/7)
	Hispanic	10.4 (5/48)	8.3 (1/12)	7.7 (1/13)	18.8 (3/16)	0.0 (0/7)
	White	72.9 (35/48)	66.7 (8/12)	69.2 (9/13)	68.8 (11/16)	100.0 (7/7)
	Other	8.3 (4/48)	16.7 (2/12)	7.7 (1/13)	6.3 (1/16)	0 (0/7)
BMI	Normal	22.9 (11/48)	16.7 (2/12)	7.7 (1/13)	43.8 (7/16)	14.3 (1/7)
	Overweight	22.9 (11/48)	16.7 (2/12)	38.5 (5/13)	6.3 (1/16)	42.9 (3/7)
	Obese	54.2 (26/48)	66.7 (8/12)	53.8 (7/13)	50.0 (8/16)	42.9 (3/7)
Gestational age	Preterm	18.8 (9/48)	0 (0/12)	15.4 (2/13)	25.0 (4/16)	42.9 (3/7)
Mode of delivery	Cesarean	52.1 (25/48)	50.0 (6/12)	61.5 (8/13)	43.8 (7/16)	57.1 (4/7)
Fetal growth	Restricted	4.2 (2/48)	0 (0/12)	0 (0/13)	6.3 (1/16)	14.3 (1/7)

### Relationship between chronic hypertension, SF, and SI

**Fig. 2:**
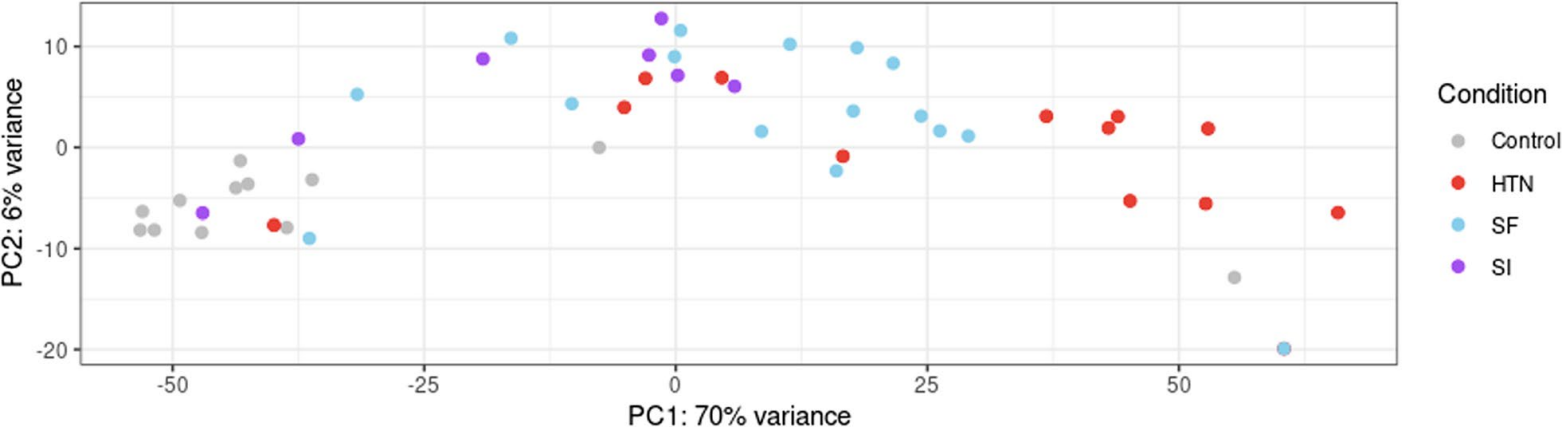
Principal component analysis of sample read counts from participants with no-hypertension (controls), chronic hypertension (HTN), preeclampsia with severe features (SF), and preeclampsia superimposed on hypertension (SI). Two dimensions of variance were plotted, PC1 on the X-axis and PC2 on the Y-axis, using the top 500 transcripts with the most variance across samples

In the analysis comparing normotensive controls to the hypertensive disease states, 2,290 genes were differentially expressed. Principal component analysis of the read counts for these genes demonstrated potential contrasts between the control samples and samples from individuals with chronic hypertension without superimposed preeclampsia (Fig. 2). Principle component analysis also showed differences in gene expression between placentas from pregnant people with SF versus SI, chronic hypertension versus SF, and chronic hypertension versus SI respectively (Fig. 2).

**Fig. 3:**
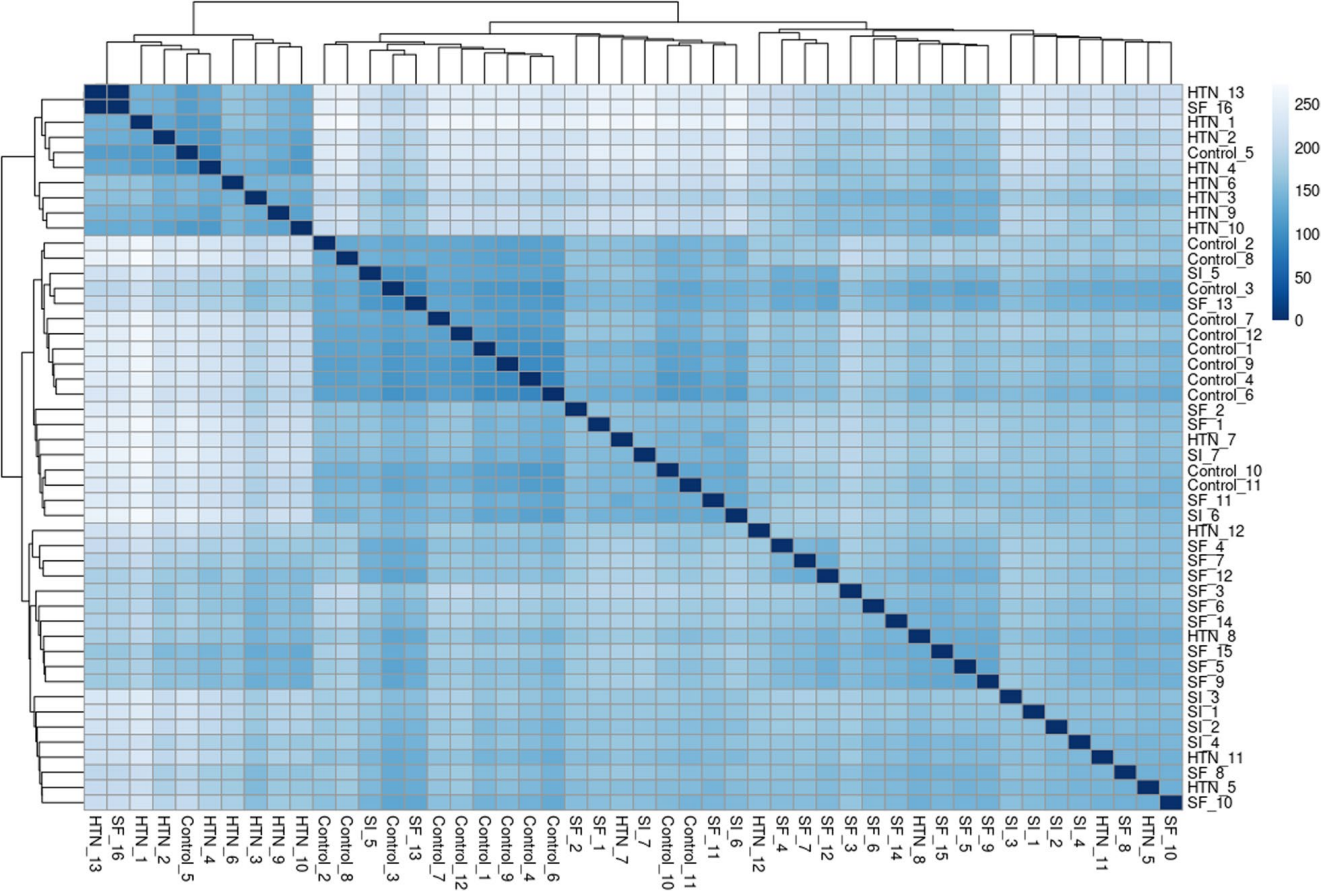
Heat map of Euclidean sample distances by hypertensive disease state for transcripts with read counts > 10. Individual samples were numbered within each disease state: participants with no hypertension (controls), chronic hypertension (HTN), preeclampsia with severe features (SF), and preeclampsia superimposed on hypertension (SI)

The differences between samples from pregnant people with chronic hypertension, SF, and SI were illustrated by way of hierarchical heat map (Fig. 3) and log-fold change correlation. As demonstrated in Fig. 3, the sample distances for HTN clustered together, as did the sample distances for SF and SI. Correlation in log2-fold changes for individual genes were used to further compare and contrast each disease state. Only term and preterm SF had correlation coefficients greater than 0.5 with chronic hypertension (R = 0.59 for term SF vs chronic hypertension and R = 0.63 for preterm SF vs chronic hypertension). SI had correlation coefficients < 0.3 with chronic hypertension for said curves (R = 0.21 for term SI and 0.22 for preterm SI). Likewise, preterm SI and preterm SF had a correlation coefficient of 0.20, while term SI and term SF had a correlation coefficient of 0.31.

### Relationship between controls and hypertensive disease states (SI, SF, HTN)

553 of the 2290 total differentially expressed genes (24.1%) were downregulated in hypertensive states relative to non-hypertensive controls, while 1,737 (75.1%) were upregulated. The majority of genes that were significantly up or down regulated were identified in the contrast between chronic hypertension and non-hypertensive controls (N = 2053, 89.7%). Preterm SI and term SI had 127 and 12 differentially expressed genes respectively in their comparisons with non-hypertensive controls. Preterm SF had 43 and term SF had 54 (see Additional file 1: Item 4).

**Fig. 4:**
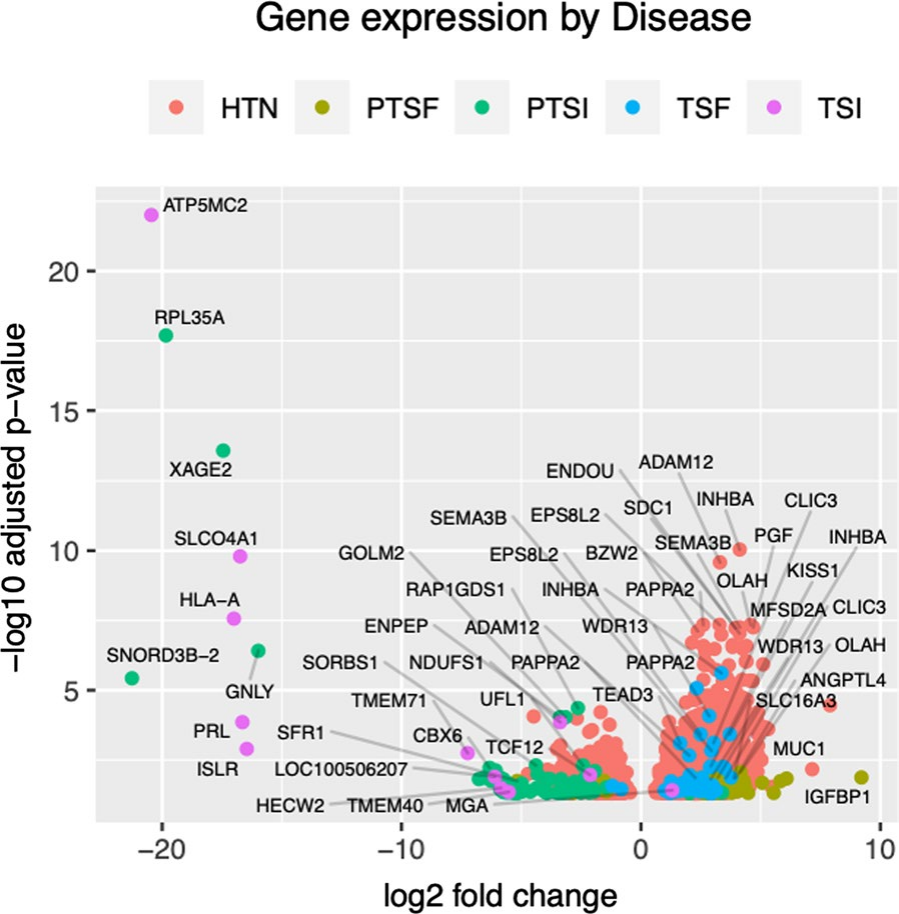
A volcano plot showing significantly upregulated (positive log2-fold change) and downregulated (negative log2-fold change) genes by condition. *HTN* Hypertension, *PTSF* Preterm PreE with severe features, *TSF* Term PreE with severe features, *PTSI* Preterm SI, *TSI* Term SI

As shown in Fig. 4, the genes that were differentially expressed in term and preterm SI were largely downregulated, while the genes involved in term and preterm SF were predominantly upregulated. For SI, 92.1% (N = 128) of the genes were significantly downregulated in comparison to controls. Conversely, SF, 91.8% (N = 97) of the genes were significantly upregulated (Additional file 2: Item 6).

### Annotation of differentially expressed transcriptomes

**Fig. 5:**
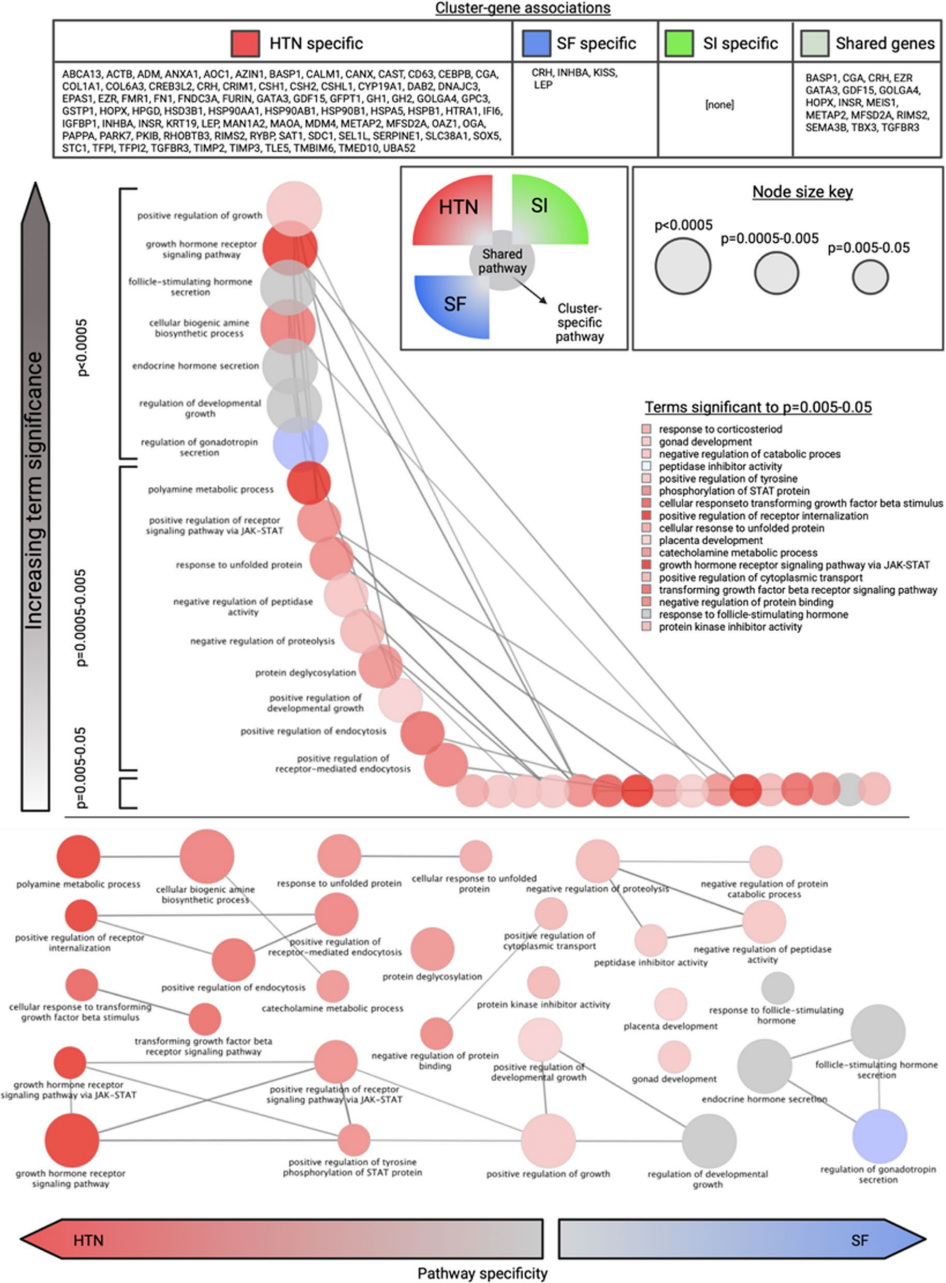
Gene ontology results by condition cluster (*HTN* Hypertension, *SF* Preeclampsia with severe features, *SI* Superimposed preeclampsia) and Bonferroni-corrected significance. Node size represents significance. Condition- “specific” genes refer to those driving identification of biologic pathways in the ClueGO gene ontology. Pathways were shaded as red for HTN, green for SF, and blue for SI based on the number of pathway-associated genes significantly differentially expressed in a given condition relative to controls

A gene ontology was created for the overall sample and subdivided into clusters representing SI, SF, and HTN, as visualized in Fig. 5. The functions that were most closely associated with HTN were cellular metabolic processes and growth regulation. SI did not have any uniquely identified pathways, instead the differentially expressed genes in this condition were associated with a subset of the pathways linked to HTN. SF, by contrast, had an overrepresentation in pathways that regulate hormone response. “Placental development” was a significant pathway shared by the three conditions of interest.

**Fig. 6:**
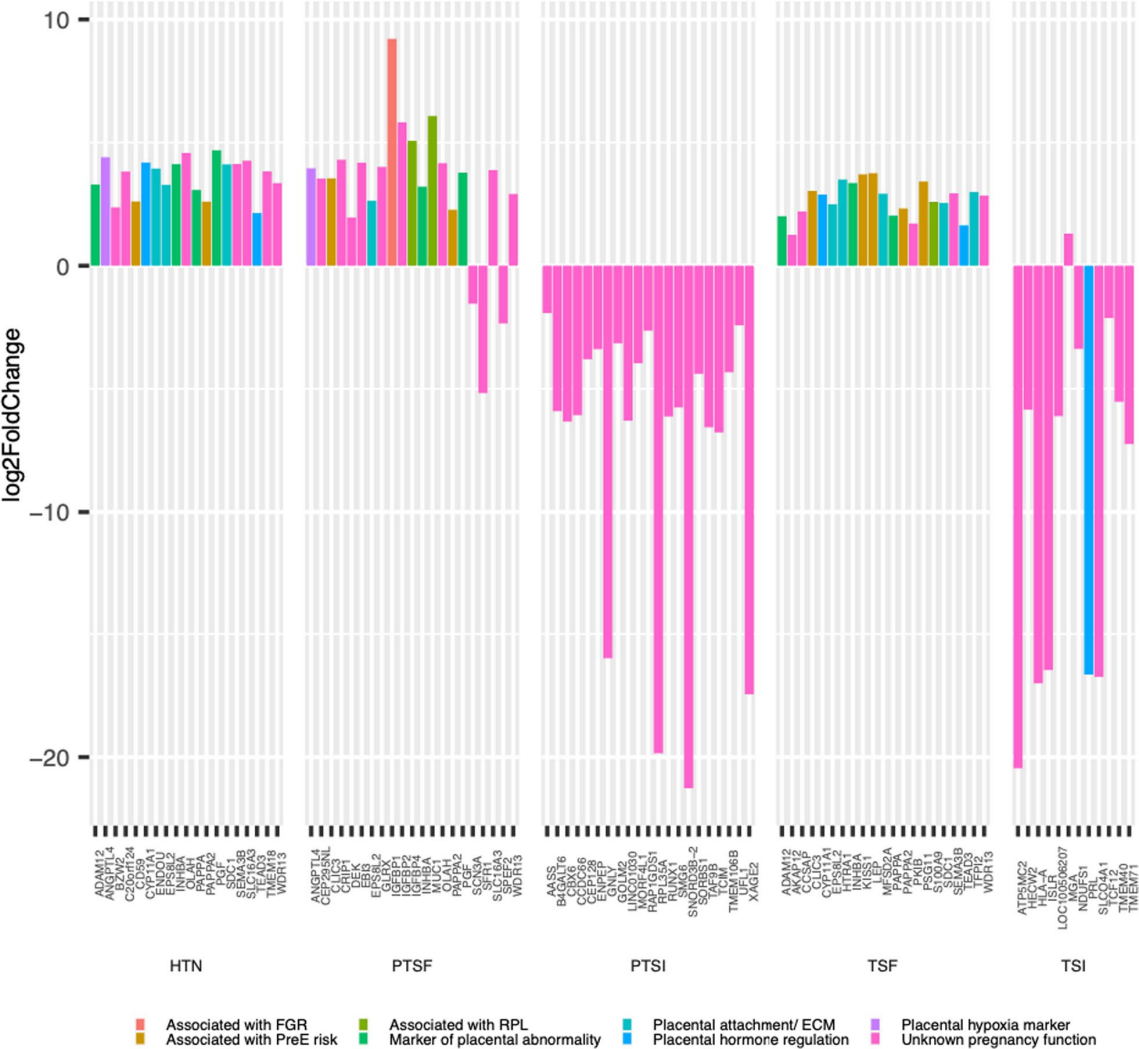
Log2-fold changes in the top twenty most significantly differentially expressed genes in each condition-gestational age grouping (*HTN* Hypertension, *PTSF* Preterm PreE with severe features, *TSF* Term PreE with severe features, *PTSI* Preterm SI, *TSI* Term SI) when contrasted with controls. Each differentially expressed gene was annotated with its described function in pregnancy or lack thereof (*RPL* Recurrent pregnancy loss, *FGR* Fetal growth restriction, *ECM* Extracellular matrix). These functional assignments were based on manual review of available data in the DAVID database as well as cited references

The results of the manual functional analysis focusing on pregnancy-related functions provided additional detail with respect to reproductive pathways represented by the differentially expressed genes. The pregnancy-related functions of the twenty most significant differentially expressed genes were manually reviewed and shown in Fig. 6. Only one of the genes in either preterm or term SI had a function that is central to reproductive processes, prolactin. Prolactin, a marker of decidualization in the placenta [8], was downregulated in term SI relative to controls. The other affected genes in SI had a wide range of documented functions both in and outside of pregnancy: roles in immune regulation (e.g., HLA), cell cycle regulation (e.g., CBX6), and metabolism (e.g., IGFBP1). The genes involved in SF, however, had many pregnancy-specific functions. They were described as markers of placental abnormality (e.g., INHBA, ADAM12, PGF), preeclampsia risk (e.g., PAPPA2, KISS1, CLIC3), and placental hypoxia (e.g., ANGPTL4). Several of the genes with well-described roles in pregnancy which were differentially expressed in SF were dysregulated in chronic hypertension as well (e.g., TEAD3, SDC1, PAPPA2, INHA, PGF).

**Fig. 7:**
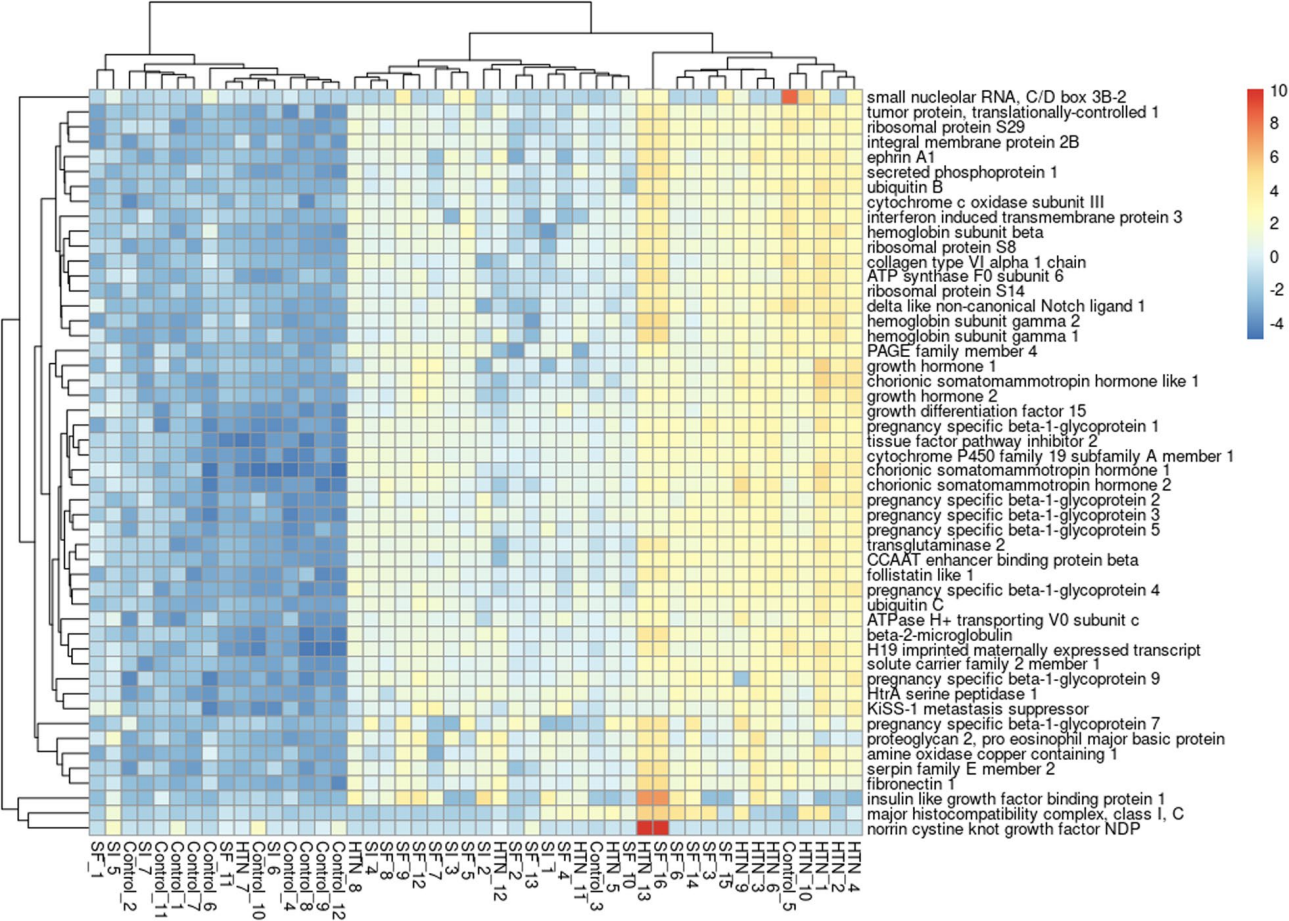
Log transcript counts of the 50 genes with the most variance in the sample, with full gene names for corresponding transcripts. The counts are given by sample, where the samples were labeled by disease state. *HTN* Hypertension, *SF* Preeclampsia with severe features, *SI* Superimposed preeclampsia

When overall expression was considered, as opposed to log-fold change relative to control samples, similar functional generalizations emerged for each hypertensive disease state. Upregulation of pregnancy related transcripts such as KISS1 and PSG1 as well as hormone regulators (e.g., CSH1) characterized HTN and SF samples, while the same genes are relatively downregulated in SI samples (Fig. 7). Though there was some overlap between SI and SF patterns of expression as suggested by other analyses reported here, genes with known proliferative roles in placental tissue had decreased expression in SI.

## Discussion

The present work analyzed the transcriptomes of placental samples from patients affected by PreE with severe features (SF) and PreE superimposed on chronic hypertension (SI) with respect to normotensive and chronically hypertensive controls. The distribution and functions of differentially expressed genes were compared and contrasted across these conditions with the intent of refining our classification of SI versus preeclampsia that occurs in the absence of baseline hypertension.

The distribution of gene expression in SI versus SF was notably contrastive, where SF was more similar to chronic hypertension than SI. The genes identified in SF were largely known markers of abnormal placentation, the majority of which were upregulated. This is consistent with prior RNA sequencing studies of preeclamptic placentas, wherein greater than 85% of differentially expressed genes were upregulated. The specific genes and pathways identified for SF and HTN in the present study are also in line with the existing literature [10, 11].

Inhibin A/B [12–14], PAPPA2 [15, 16], ADAM12, and KISS1 upregulation for example, have been recognized in multiple studies of pathologic placentation and hypertensive pregnancies [17]. The inhibin family of proteins positively regulate placental trophoblastic invasion through their SMAD2/3-SMAD4 dependent modulation of N-cadherin [18]. At high concentrations however, they inhibit the production of metalloproteases necessary for remodeling of the decidua, thus contributing to the pathologic placentation observed in preeclampsia. KISS1 has a similarly described mechanism wherein it inhibits metalloproteases and disrupts physiologic decidual invasion [19]. Both ADAM12 and PAPPA2 are insulin-like growth factor-binding protein proteases hypothesized to be downstream products of this disrupted decidualization, possibly upregulated by the associated relative hypoxia [20]. PGF and ANGPTL4 have recognized roles in hypertensive pregnancy as well, mediating angiogenesis. Though the serum levels of their protein products are typically decreased in patients with preeclampsia [16], upregulation at the placental level may be compensatory for the relative overabundance of antiangiogenic factors [21, 22].

In contradistinction to SF, SI was characterized as a loss of function process, where the majority of the significant genes were downregulated compared to normotensive controls. Though the identified genes and their associated processes are certainly involved in pregnancy, there was notably very limited overlap between the genes that were downregulated in the SI samples and genes previously implicated in pathologic conditions of pregnancy. The gene with the most specific pregnancy-related function in SI was prolactin, which showed 993-fold downregulation in term SI compared to control samples. Prolactin downregulation in placental tissue is associated with poor decidualization and has been shown to be inversely correlated with sFLT-1 levels [17]. However, the antiangiogenic and hypoxic factors that were significant in the analyses of SF and HTN were not seen in SI. These results corroborate the recent suggestion that angiogenic dysregulation, while present, is a less prominent feature in the pathogenesis of SI [23].

The genes that were downregulated in SI were associated with basic cell processes of transport, signaling, and migration as well as hormone responsiveness and immune functions. Immunologic factors and those related to cellular invasion and proliferation have been identified as contributors to PreE through genomic and transcriptomic studies [24]. These functions overlap substantially with the PreE subtypes described by Leavey and colleagues, who pose an analogy between the disruptions seen in neoplasia with the abnormal placentation described in preeclampsia [25]. Signaling and transport functions are also key placental processes whose downregulation is notable and potentially pathogenic. Dysfunctional protein kinase A signaling, one of the pathways identified in the SI gene ontology analysis, has been associated with a decrease in vasoactive growth factors [26].

Our results support a molecular basis for the heterogeneity of PreE, likely consisting of multiple clinically relevant subtypes [24–28]. Further work that integrates clinical, histopathologic, and molecular phenotyping is needed to advance this evolving translational body of work. The current study’s findings indicates that SI should be distinguished in such analyses, where it has typically been grouped with SF. A large part of the diagnostic uncertainty that plagues SI is attributable to a dearth of knowledge as to the pathophysiology of this condition and the extent to which it is or is not consistent with PreE. Our finding that SI patterns distinctly from chronic hypertension and SF at the molecular level suggests the potential for more nuanced approaches to clinical stratification of PreE sub-types. For example, it has already been demonstrated that pregnant people who have had PreE in prior pregnancies are not at a greater risk for SI in future pregnancies complicated by chronic hypertension [29]. This result is congruent with a taxonomy that considers SI and PreE in the absence of baseline hypertension to be different pathologies entirely. It may be the case that as more precise approaches to PreE evolve, unique sets of biomarkers, preventative therapies, and interventions could be proposed for SI and SF.

The research presented here has strengths in its design, sampling, and functional analysis. It is based on a relatively large cohort of pregnant people with in-depth clinical characterization. Furthermore, its experimental groups highlight clinically relevant, understudied subpopulations. This work applies both a clinical and integrative lens to its findings, identifying pathways for future research and potential clinical application. Limitations of the study include its sole focus on placental tissue. Further, conflation of pathologic versus adaptive placental processes is a known complication of such work. For instance, the upregulation of PGF and ANGPTL4 in our transcriptional analysis highlight this ambiguity. The limitations specific to placental analysis are magnified in the smaller subsets of our data. Notably, our smallest clinical subset is superimposed preeclampsia, wherein there may be considerable transcriptomic heterogeneity. Furthermore, the appropriate controls for placental studies in preeclampsia remans a controversial issue. Though no substantive differences were noted between the term and preterm groupings in this study, the controls studied here were from term deliveries to avoid introducing placental derangements associated with spontaneous preterm birth. Fetal sex was also unavailable for analysis in this study. Prospective studies that recruit carefully selected cases of iatrogenic delivery in the early preterm period for non-placental pathology (e.g., monochorionic twins) may be able to create a more optimal comparison group.

## Conclusions

Preeclampsia superimposed on chronic hypertension and preeclampsia with severe features have unique patterns of placental gene expression. These conditions are likely to be sufficiently distinct to warrant being studied individually in future research, as further investigation of these heterogeneous subgroups of preeclampsia may have divergent markers of disease severity, progression, and persistence. These considerations are important to dissect to achieve future precision health approaches to hypertension in pregnancy.

## Supplementary Information

**Additional file 1.** Supplemental Items 1–5, figures pertaining to data quality assessment and processing.

**Additional file 2.** Supplemental Item 6, a list of all differentially expressed genes with P-values < 0.05.

## Data Availability

The transcriptomic data is available in GEO, Accession ID GSE204835.
