# Supplementary material for: Placental transcriptome analysis of hypertensive pregnancies identifies distinct gene expression profiles of preeclampsia superimposed on chronic hypertension
Source: BMC Med Genomics. 2023 May 2;16:91. doi: 10.1186/s12920-023-01522-x (PMC10152005; doi:10.1186/s12920-023-01522-x)
Supplement: Supplementary file 1 — Additional file 1. Supplemental Items 1–5, figures pertaining to data quality assessment and processing. [file 12920_2023_1522_MOESM1_ESM.docx]

**Supplemental materials item 1**

Per Sequence Quality Scores


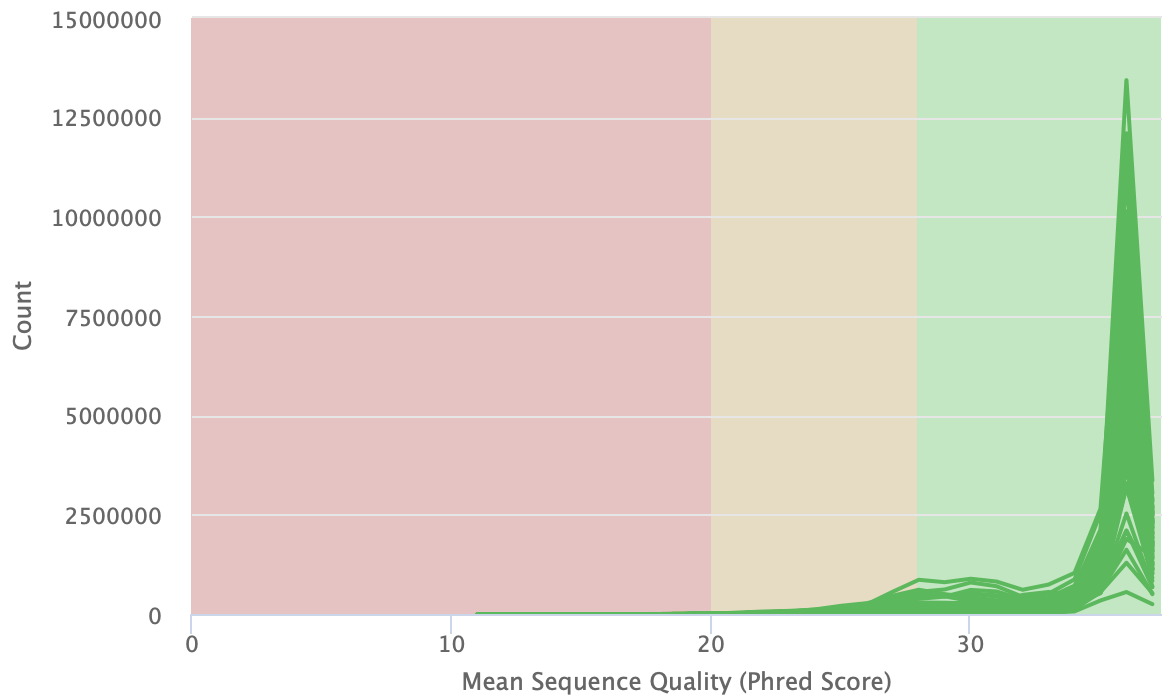


The number of reads by average quality scores.

**Supplemental materials item 2**

PCA of patients who endorsed smoking in the pregnancy (“Smoker”) versus those who did not (“Non”).

**Supplemental materials item 3**

PCA of patients with a history of autoimmune disease (“AI”) versus those without such history (“noAI”).

**Supplemental materials item 4**

Heatmap of all files available for analysis.

**Supplemental materials item 5**


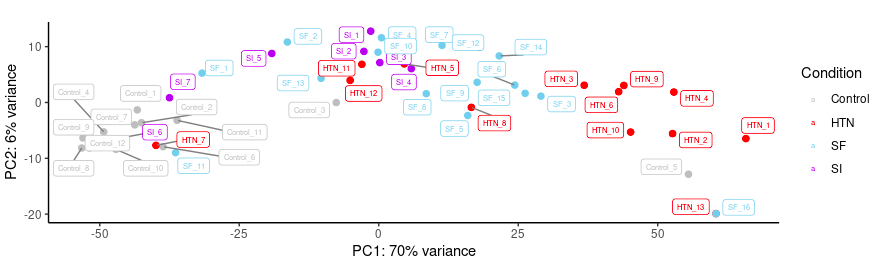


Sample numbering represents order of collection within each condition (e.g., HTN_1 was the first HTN sample collected, HTN_12 was the last HTN sample collected).
